# Seeking Polymeric Prodrugs of Norfloxacin. Part 2. Synthesis and Structural Analysis of Polyurethane Conjugates

**DOI:** 10.3390/molecules15020842

**Published:** 2010-02-05

**Authors:** Marcin Sobczak, Katarzyna Nurzyńska, Waclaw Kolodziejski

**Affiliations:** Department of Inorganic and Analytical Chemistry, Faculty of Pharmacy, Medical University of Warsaw, ul. Banacha 1, 02-097 Warsaw, Poland; E-Mails: katarzyna.nurzynska@op.pl (K.N.); waclaw.kolodziejski@wum.edu.pl (W.K.)

**Keywords:** aliphatic polyesters, aliphatic polyurethanes, ring-opening polymerization, macromolecular conjugates, prodrugs of norfloxacin

## Abstract

Oligo(ε-caprolactone) and oligolactide were synthesized via ring-opening polymerization of cyclic esters in the presence of creatinine as initiators. Thus obtained oligomers were successfully used in the synthesis of novel polyurethane conjugates of norfloxacin. The structures of the polymers and conjugates were elucidated by means of MALDI-TOF MS, NMR and IR studies.

## 1. Introduction

Pharmacy is one of the most important areas of applications of polymers. They are used as active macromolecular pharmaceutical substances, blood substitutes, auxiliary materials and excipients, reagents for synthesis of macromolecular prodrugs, in polymeric drug delivery systems, therapeutic systems, *etc*. [1,2,3,4,5,6,7,8,9,10,11]. Aliphatic polyurethanes have good biodegradability and biocompatibility properties. These attributes make them extremely useful in medical and pharmaceutical industries, for example to produce implants containing controlled drug delivery systems [12,13].

Aliphatic polyurethanes are usually prepared by reaction of aliphatic diisocyanate with compounds having two reactive hydrogen atoms (oligomers) reaction. Alternatively, aliphatic polyurethanes can be prepared by non-isocyanate methods, if five-membered cyclic carbonate groups are reacted with diamines. Aliphatic polyurethanes can be synthesized by ring-opening polymerization of cyclic urethane, too [14,15,16,17,18]. Polyesters, polyethers and polycarbonates are usually used as oligomers. They were successfully synthesized by ring opening polymerization of cyclic monomers in the presence of cationic or anionic initiators, as well as coordinating and enzymatic catalysts [19,20,21,22,23,24,25,26,27,28,29,30,31]. Recently, Wang has found that creatinine, a non-toxic metabolite in the human body, shows rather satisfactory catalytic properties for polymerization of L-lactide [32]. Creatinine is a break-down product of creatine phosphate in the muscle tissue, and is usually produced in the body at a fairly constant rate (depending on the muscle mass). Chemically, creatinine is a spontaneously formed, cyclic derivative of creatine. Creatinine is chiefly filtered out of the blood by the kidneys. 

Fluoroquinolones comprise an important new class of synthetic oral antibacterial agents used against various infections. They efficiently kill bacteria and/or prevent their growth. The first discovered and clinically effective quinolone was norfloxacin, that is 1-ethyl-6-fluoro-1,4-dihydro-4-oxo-7-(1-piperazinyl)-3-quinolinecarboxylic acid [33]. Recently, the interaction of norfloxacin with DNA has been considered as useful in the anticancer drug design [33]. Preliminary efforts to prepare nanoparticles of poly(D,L-lactide-co-glycolide) loaded with adsorbed norfloxacin has already been reported [34]. The anticancer action of norfloxacin is supposed to be improved by anchoring the drug, using a chemical linkage, to biodegradable oligomers. Such delivery systems can transport the drug molecules more efficiently and more specifically. We would like to follow the latter scientific direction.

Controlled drug delivery technology represents one of the most rapidly advancing areas of science. The polymeric prodrugs, drug delivery systems and therapeutic systems exhibit unique pharmacokinetics, body distribution and pharmacological efficacy [1,2,3,4,5,6,7,8,9,10,11]. Recently, the polyester prodrugs of norfloxacin were obtained in our laboratory [35]. Two-, three- and four-arm, star-shaped poly(ε-caprolactone) and poly(D,L-lactide) homopolymers, and copolymers of ε-caprolactone with D,L-lactide were synthesized via ring-opening polymerization of cyclic esters in the presence of glycerol, penthaerythritol and poly(ethylene glycol) as initiators and stannous octoate as a catalyst. Thus obtained oligomers were successfully used in the synthesis of novel macromolecular prodrugs of norfloxacin [35].

In this paper, we describe the synthesis of a series of linear polyurethane conjugates of norfloxacin. Chemical structures of the synthesized polymers have been confirmed using ^1^H- and ^13^C- solution NMR and FT-IR spectroscopy. Molecular weights of polymers have been determined using gel permeation chromatography (GPC) and a viscosity method. The present paper is the continuation of our previous work [35]. We believe that the obtained polyurethanes can find practical applications as effective drug delivery systems transporting active substances to specific body locations at the required rate.

## 2. Results and Discussion

### 2.1. Ring-opening polymerization of cyclic esters

The first test of L-lactide (L-LA) polymerization in the presence of creatinine (CE) was described by Wang [32]. Thus obtained poly(L-lactide) (PLLA), terminated by hydroxyl and carboxyl groups, had M_n_ = 6,700–14,000 Da and narrow polydispersity (PD = 1.20–1.57). We have decided to carry out more detailed investigation of that reaction by extending the range of monomers to ε-caprolactone (CL) and rac-lactide (D,L-LA). The aim was to obtain a low-molecular weight polyesters, terminated at both sides by hydroxyl groups, which can be subsequently used as segments in polyurethane conjugates. 

**Scheme 1:**
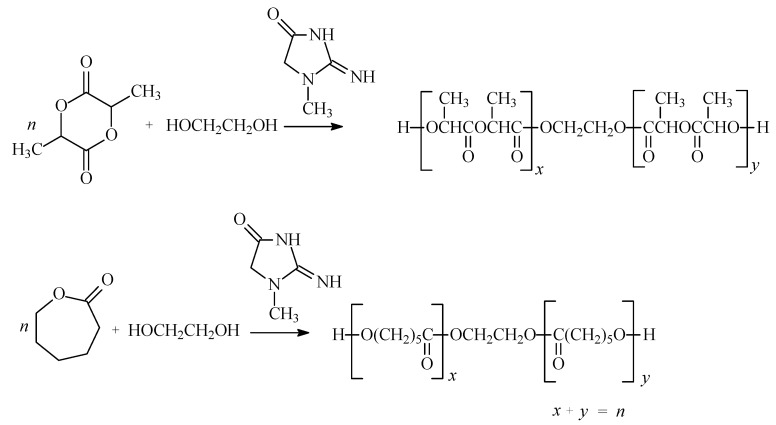
The synthesis scheme of oligoesters.

**Table 1:** The homopolymerization of CL, LA and D-LA, initiated by CE.

Run no.	Monomer	Molar ratio^*^	Yield [%]^**^	Physical form^***^	M_n_^MALDI^ [Da]	PD ^MALDI^	M_n_^GPC^ [Da]	PD^GPC^	M_LOH_ [Da]
1	CL	100:1:1	63%	ss	-	-	4,900	1.3	-
2	CL	50:1:1	72%	ss	-	-	3,600	1.2	2,400
3	CL	25:1:1	91%	ss	2,700	1.2	3,100	1.3	2,600
4	L,L-LA	100:1:1	42%	vl	-	-	4,800	1.3	-
5	L,L-LA	50:1:1	51%	vl	1,400	1.2	3,100	1.2	2,000
6	L,L-LA	25:1:1	62%	vl	-	-	2,200	1.2	-
7	D,L-LA	100:1:1	46%	vl	2,300	1.1	-	-	-
8	D,L-LA	100:1:1	46%	vl	2,300	1.1	-	-	-
9	D,L-LA	25:1:1	64%	vl	1,600	1.1	2,200	1.2	1,800

Reaction conditions: temp. of 160 °C, time of 96 h; ^*^ monomer : CE : EG; M_n_^MALDI ^– number-average molecular weight determined by MALDI-TOF; PD ^MALDI ^- polydispersity (M_w_/M_n_) determined by MALDI-TOF; M_n_^GPC ^– number-average molecular weight determined by GPC; PD ^GPC^ - polydispersity (M_w_/M_n_) determined by GPC; ^**^ - calculated by the weight method; ^*** ^ss – sticky solid, vl – viscous liquid; M_v_ – average molecular weight from the viscosity measurements; M_LOH_ - number-average molecular weight calculated from LOH.

The polymerization reactions of CL, D,L-LA and L-LA in the presence of CE and ethylene glycol (EG) were carried out at 160 ºC for 96 h. Under these conditions the cyclic monomers underwent ring‑opening polymerizations to give low-molecular weight polyesters terminated by hydroxyl chain-end groups (Scheme 1, Table 1).

**Figure 1:**
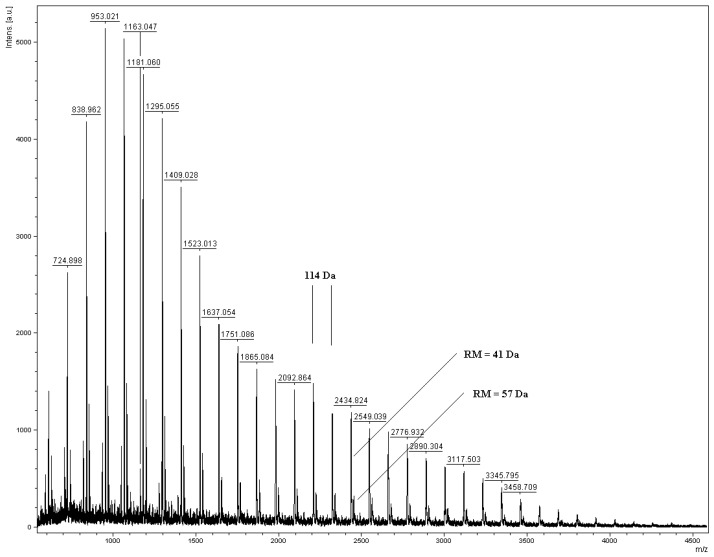
The MALDI TOF spectrum of the product of the CL polymerization in the presence of CE (Table 1, run no. 3).

**Figure 2:**
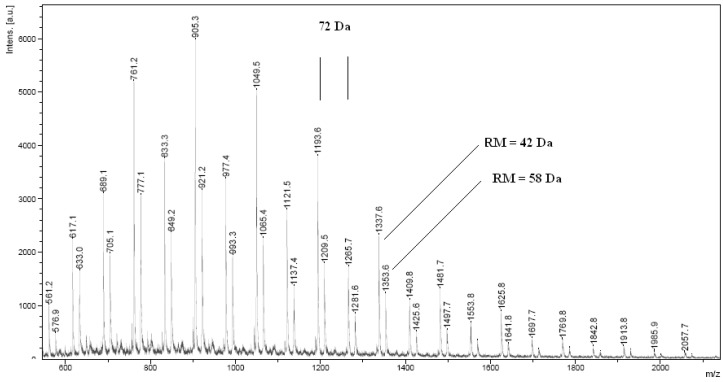
The MALDI TOF spectrum of the product of L-LA polymerization in the presence of CE (Table 1, run no. 9).

The chemical structures of the obtained polymers were confirmed by ^13^C-, ^1^H-NMR and IR studies. Figure 1 and Figure 2 show typical MALDI-TOF spectra of the obtained products.

The MALDI-TOF spectrum of PCL comprises two series of peaks. The most prominent series of peaks is characterized by a mass increment of 114 Da, which is equal to the mass of the repeating unit in the PCL polymer (Figure 1). This series is assigned to PCL terminated with a hydroxyl group and detected as the Na^+^ adduct (residual mass: RM = 41 Da). The second series of the peaks is also from PCL terminated with a hydroxyl group, but corresponds to the K^+^ adduct (RM = 57 Da).

The MALDI-TOF spectrum of PLA comprises two series of peaks, too (Figure 2). The main series comes from PLA terminated with a hydroxyl group and corresponds to the Na^+^ adduct (RM = 42 Da), while the second series of smaller peaks is also from PLA terminated with a hydroxyl group, but corresponds to the K^+^ adduct (RM = 57 Da). In the MALDI-TOF spectrum of PLA both populations of chains of even and odd number of lactic acid m.u. can be observed. The odd number of acid m.u. shows that under the process conditions the polymer chain undergoes intermolecular transesterification (leading to an exchange of segments), which is a typical phenomenon for the polymerization of lactides [20]. Formation of PCL and PLA macrocycles was not observed.

The number-average molecular weights determined from GPC for CL oligomers lie in the 3,100–4,900 Daltons range, and the polydispersity indexes in the 1.2–1.3 range. For L-LA and D,L-LA oligomers the M_n_ values are 2,200–4,800 and the M_w_/M_n _values lie in the 1.2–1.3 range. The M_n_ values determined by the GPC method differ by 16–35% from those determined by the conventional method of the terminal group analysis (Table 1). Similar differences occur between the M_n_ values determined on the basis of the hydroxyl number and those obtained from the MALDI TOF experiments. However, the MALDI TOF work is still insufficient yet to judge whether this method can be used for quantitative determination of the end group concentration.

The mechanism of the ring-opening polymerization of L-LA in the presence CE was proposed by Wang [32]. The coordination-insertion mechanism was postulated. The kinetic and mechanistic studies of the polymerization of cyclic monomers in the presence of CE are underway in our laboratory, too. The results are to be presented in next papers.

### 2.2. Synthesis of polyurethane conjugates of norfloxacin

**Scheme 2:**
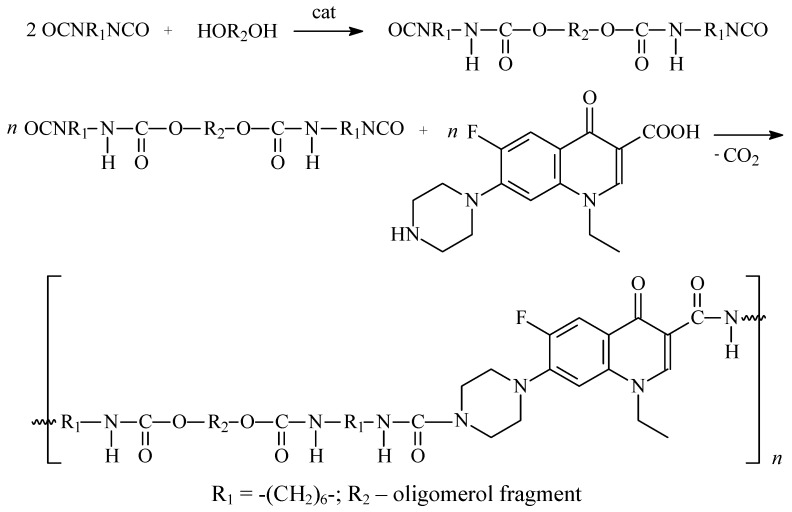
The synthesis scheme of polyurethane conjugates (two-step process).

The macromolecular conjugates were obtained (Scheme 2) in the reactions of the PCL, PLA, commercial oligo(ethylene adipate)diol (OEAD) and oligo(ε-caprolactone)diol (OCL) with 1,6-hexane diisocyanate **(**HDI) and norfloxacin (NOR). Polyurethanes were synthesized in one and two-step processes.

As polyurethane segments, we used PCL diols of M_n_ = 2,400 and 2,600 Da (Table 1), PLA diols of M_n_ = 1,800 and 2,000 Da, and commercially available OEAD of M_n_ = 1,000 Da and OCL of M_n_ = 2,000 Da. The HDI:oligodiol:NOR molar ratio was always 2:1:1, which corresponds to a so called isocyanate index equal to 1. Stannous octoate (SnOct_2_) or dibutyltin dilaurate (DLDBSn) were applied as the polyaddition catalysts. The reaction conditions and molecular weight of the obtained products are listed in Table 2. We found that the synthesized by us and commercial polyesters diols underwent polyaddition giving polyurethanes of M_v_ = in the range of 14,200–42,800 Da, as determined using the viscosity method. The degree of polyaddition was dependent on the kind of catalyst used. Then, in all the systems studied, the polyurethanes obtained in the presence of DLDBSn were characterized by higher molecular weights than those prepared using SnOct_2_. 

**Figure 3:**
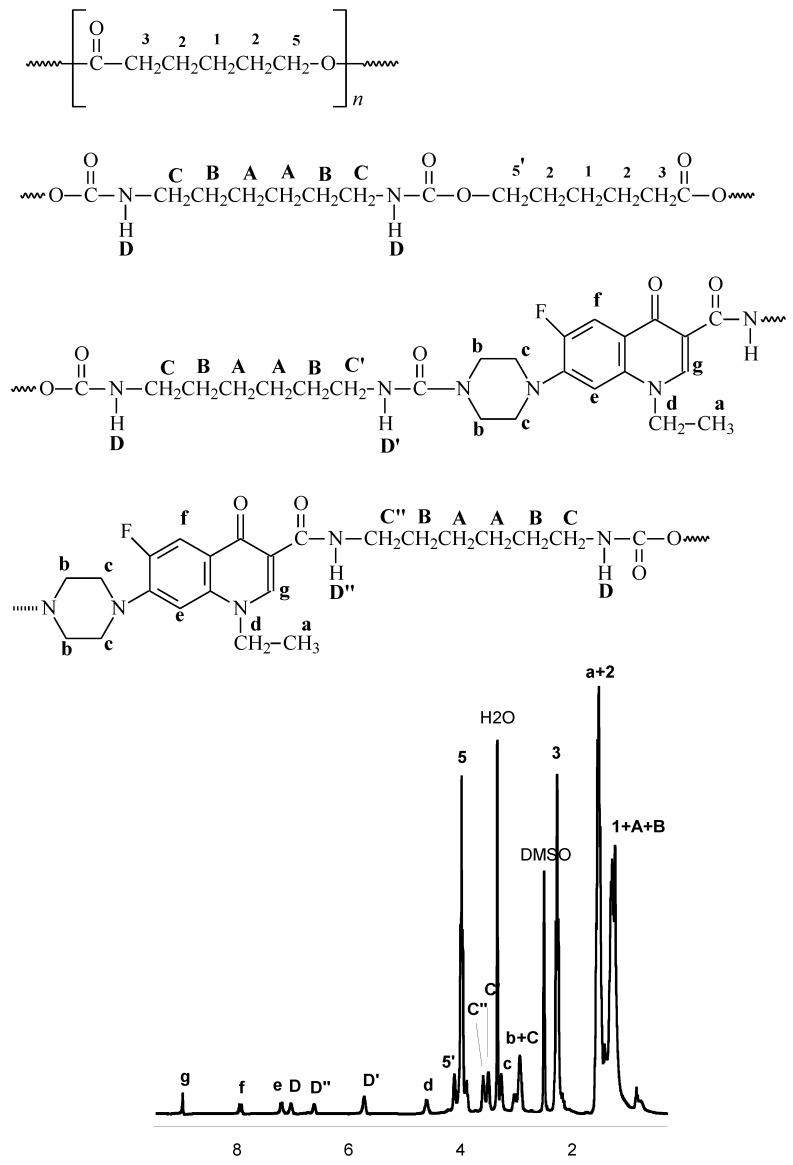
The ^1^H-NMR spectrum of the conjugate: NOR-PUR (PCL-HDI) (in DMSO-d_6_) (Table 2, Run no. 11).

The chemical structures of the prepared polymers were confirmed by ^13^C, ^1^H-NMR and IR studies. Figure 3 presents typical ^1^H-NMR spectrum of the obtained polyurethane conjugate.

**Figure 4:**
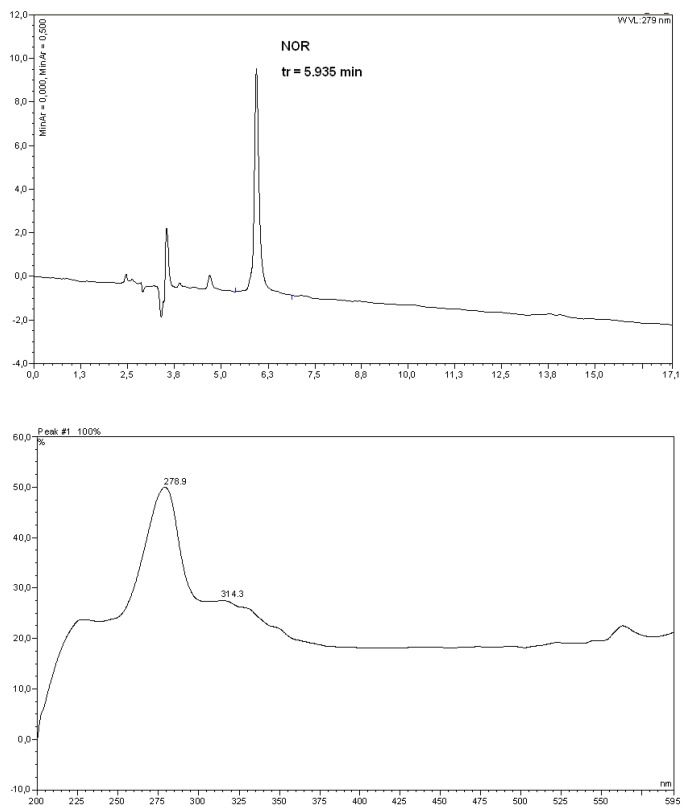
The HPLC chromatogram and UV spectrum of norfloxacin released from polyurethane conjugates (Table 3, Run no. 3, degradation in phosphate buffer, after 21 days).

Preliminary studies on the release of the norfloxacin from polyurethane conjugates have made. It has turned out that the drug is gradually released from the obtained polyurethane conjugates (Figure 4). 

**Table 2:** Polyurethane conjugates of norfloxacin.

Run no.	Reagents/catalyst	Synthesis methods^*^	Yield [%]^**^	M_v_ [Da]
1	HDI-OEAD-NOR DLDBSn	II	≈ 100	23 600
2	HDI-OEAD-NOR DLDBSn	I	≈ 95	20 500
3	HDI-OEAD-NOR SnOct_2_	I	≈ 92	18 900
4	HDI-OEAD-NOR SnOct_2_	I	≈ 88	14 200
5	HDI-OCL-NOR DLDBSn	II	≈ 96	33 300
6	HDI-OCL-NOR DLDBSn	I	≈ 93	26 200
7	HDI-OCL-NOR SnOct_2_	II	≈ 86	30 300
8	HDI-OCL-NOR SnOct_2_	I	≈ 79	21 400
9	HDI-PCL1-NOR DLDBSn	II	≈ 92	40 900
10	HDI-PCL1-NOR SnOct_2_	II	≈ 84	30 700
11	HDI-PCL2-NOR DLDBSn	II	≈ 94	42 800
12	HDI-PCL2-NOR SnOct_2_	II	≈ 87	33 300
13	HDI-PLA1-NOR DLDBSn	II	≈ 79	24 200
14	HDI-PLA1-NOR SnOct_2_	II	≈ 65	20 200
15	HDI-PLA2-NOR DLDBSn	II	≈ 72	18 400
16	HDI-PLA2-NOR SnOct_2_	II	≈ 59	14 700

Reaction conditions: temp. 65 °C, time – 3h (for the one-step process) or 6h (for the two-step process), molar ratio HDI: macrodiol: NOR: catalyst = 2:1:1:0.01; PCL1 (M_n_= 2,600 Da), PCL2 (M_n_= 2,400 Da), PLA1 (M_n_= 2,000 Da), PLA2 (M_n_= 1,800 Da); ^*^ I – one-step process, II – two-step process; M_v_ – average molecular weight from the viscosity measurements; ^**^ - calculated by the weight method.

Table 3 shows the degree of norfloxacin released from of the selected polyurethane conjugates as function of time under mild conditions in HCl buffer (pH=1) and phosphate buffer (pH=7,4), respectively.

**Table 3:** Release of the norfloxacin from polyurethane conjugates.

Run no.	Reagents/catalyst	C	pH = 1			pH = 7.4		
			P^7^	P^14^	P^21^	P^7^	P^14^	P^21^
1	HDI-OEAD-NOR DLDBSn	19	5	9	16	3	5	7
2	HDI-PCL1-NOR DLDBSn	17	4	6	10	2	3	5
3	HDI-PLA1-NOR DLDBSn	18	6	11	18	4	7	10

C - NOR units content in the polyurethane conjugates (% mol), P^7^ - percent of norfloxacin released from the polyurethane conjugates after 7 days, P^14 ^– after 14 days, P^21 ^– after 21 days; C – calculated by ^1^H NMR (signal intensity of the –O(O)CNHC**H_2_**CH_2_-/signal intensity of the –NHC**H_2_**CH_3_); P – determined by UV spectroscopy method.

Preliminary results show the rate of norfloxacin release from polyurethane conjugates depends on the structure of the polymer and the order of hydrolysis is as follows: poly(lactide-polyurethane) > poly(ethylene adipate-polyurethane) > poly(ε-caprolactone-polyurethane). The results suggest a high stability of obtained polyurethane conjugates to chemical hydrolysis at pH 7,4 than 1.

Kinetics of the NOR release from polyurethane conjugates are still under study and will be presented in the next paper. We shall also discuss correlation between the structure of the polyurethane conjugates and the drug release rate. 

## 3. Experimental

### 3.1. Materials

ε-Caprolactone (CL, 2‑oxepanone, Aldrich 99%) was dried and distilled before use over CaH_2_ at reduced pressure. 3,6-Dimethyl-1,4-dioxane-2,5-dione (D,L-LA and L-LA, rac-lactide and L-lactide, Aldrich 98%) was crystallized from a mixture of dry toluene with hexane and dried under vacuum. Creatinine (CE, Aldrich 99%), norfloxacin (NOR, Aldrich 99%), ethylene glycol (EG, Aldrich, 95%), 1,6-hexane diisocyanate (HDI, Aldrich 99%), oligo(ethylene adipate)diol (OEAD, Aldrich 95%, M_n_ = 1,000 Da) and oligo(ε-caprolactone)diol (OCL, M_n_ = 2,000 Da, Aldrich 99%) were exhaustively dried under vacuum prior to use. Stannous octoate (SnOct_2_, tin (II) 2-ethylhexanoate, Aldrich 95%), dibutyltin dilaurate (II) (DLDBSn, Aldrich, technical), dichloromethane (POCh), anhydrous dimethyl sulfoxide (DMSO, Aldrich 99%) and anhydrous methanol (POCh) were used as received. 

### 3.2. Instrumentation

The polymerization products were characterized by means of ^1^H- and ^13^C-NMR (Varian 300 MHz), and FTIR spectroscopy (Spectrum 1000, Perkin Elmer). The NMR spectra were recorded in CDCl_3_ or DMSO-d_6_. The IR spectra were recorded from KBr pellets. Relative molecular mass and molecular mass distributions were determined using MALDI-TOF MS, GPC and viscosity techniques. The MALDI-TOF spectra were measured in the linear mode on a Kompact MALDI 4 Kratos analytical spectrometer using a nitrogen gas laser with 2-[(4-hydroxyphenyl)diazenyl] benzoic acid (HABA) as a matrix [the samples were dissolved in CHCl_3_ (10 mg/mL) and THF (20 mg/mL)]. The average molecular mass and the molecular mass distribution of the macromolecular conjugates were measured by means of the GPC technique (LabAlliance) at 25 °C (the samples were dissolved in chloroform). The device was calibrated with polystyrene standards.

Intrinsic viscosities of polyurethane solution in DMF were measured at 30 °C using Ubbelohde capillary viscometer (K = 0,01152). The concentrations of polymer solutions were 0.2, 0.4, 0.6, 0.8 and 1.0 vol. %. The average molecular weight from the viscosity measurement was calculated using the Mark-Houwink equation with the following constants: K = 6.80·10^-5^ dL/g and α = 0.86 [35].

The hydroxyl number of the obtained polycarbonate diols was determined according to the conventional method, based on the reaction with acetic acetate [15].

The amount of released norfloxacin was determined by a UV-Vis spectrophotometry (UV-1202 Shimadzu) at the adsorption maximum of the free drug in aqueous buffered solutions (*λ*_max_ = 279 nm) using a 1 cm quartz cell.

HPLC-analysis of Norfloxacin was performed on a Phenomenex-RPC_18_ column (250 × 4.6 mm, 5 µm) (Dionex P-580 chromatograph). The eluent was a mixture of water/acetonitrile/trifluoroacetic acid (80/20/0.1/%). The norfloxacin was spectrophotometrically detected at 279 nm. 

### 3.3. General procedure

#### 3.3.1. Oligoesters synthesis

Monomers (CL, D,L-LA, L-LA, 25 mmol), EG and CE were placed in a 10 mL glass ampule under argon atmosphere. The reaction vessel was then kept standing in a thermostated oil bath at 160 °C for 96 h (Table 1). When the reaction time was completed, the reaction product was dissolved in CH_2_Cl_2_, then precipitated from cold methanol using diluted hydrochloric acid (5% aqueous solution) and finally dried under vacuum for 7 days. The precipitation was repeated three times.

#### 3.3.2. Macromolecular conjugates synthesis

Polymeric conjugates of NOR were synthesised using both the one-step and two-step methods. In the one‑step method, at the beginning NOR was added to the mixture of oligomers, HDI and the catalyst. Then, this reaction mixture was stirred for 3 hours at 65 °C. In the two-step method, oligoestrodiols were mixed with HDI (25 mmol) in a molar ratio 1:2 and dissolved in DMSO. This solution was placed in a three-necked flask equipped with a stirrer and a thermometer. Afterwards, a catalyst was added to the flask and the reaction mixture was left with stirring for 3 hours at 65 °C. Then, the solution of NOR in DMSO was added dropwise into the reactor with the prepolymer under vigorous stirring (in the molar ratio of NOR to prepolymer equal 1:1). After the addition procedure was completed, the reaction mixture was left with stirring for additional 3 hours at 65 °C. Then, it was washed with diluted hydrochloric acid (5% aqueous solution) and water. The precipitation was repeated three times. The conjugates isolated from the solution’s organic phase were kept under vacuum at room temperature for no more than one week. 

### 3.4. Biodegradation of polyurethane conjugates

Dried polymer (20 mg) was poured into aqueous buffered solution (200 mL, pH 1 and 7.4) at 37 ºC. The mixture was stirred and a 2 mL sample was removed at selected intervals and 2 mL of buffer was replaced. The quantity of released drug was analyzed by means of chromatograph and UV spectrophotometer determined from the calibration curve obtained previously under the same conditions.

### 3.5. IR and NMR data

*PCL*: ^1^H-NMR (CDCl_3_, δ, ppm): 1.25 (2H, m, -OCH_2_CH_2_C**H_2_**CH_2_CH_2_C(O)O-), 1.66 (4H, m, ‑OCH_2_C**H_2_**CH_2_C**H**_2_CH_2_C(O)O-), 2.31 (2H, t, -OCH_2_CH_2_CH_2_CH_2_C**H_2_**C(O)O-), 3.65 (2H, t, ‑C**H_2_**OH, end group), 4.11 (2H, t, -OC**H_2_**CH_2_CH_2_CH_2_CH_2_C(O)O-); ^13^C-NMR (CDCl_3_, δ, ppm): 24.9 (‑OCH_2_CH_2_**C**H_2_CH_2_CH_2_C(O)O-), 25.8 (-OCH_2_CH_2_CH_2_**C**H_2_CH_2_C(O)O-), 28.4 (‑OCH_2_**C**H_2_CH_2_CH_2 _CH_2_C(O)O-), 33.8 (-OCH_2_CH_2_CH_2_CH_2_**C**H_2_C(O)O-), 63.9 (-**C**H_2_C(O)OH, end group), 64.4 (‑O**C**H_2_CH_2_CH_2_CH_2_CH_2_C(O)O-), 173.8 (-OCH_2_CH_2_CH_2_CH_2_CH_2_**C**(O)O-); FTIR (KBr, cm^-1^): 2,944 (ν_as_CH_2_), 2,867 (ν_as_CH_3_), 1,722 (νC=O), 1,244 (νC-O).

*PLA*: ^1^H-NMR (CDCl_3_, δ, ppm): 1.50 (3H, q, -CH(C**H_3_**)C(O)O-), 4.39 (1H, q, -C**H(**CH_3_)OH, end group), 5.17 (1H, q, -OC**H**(CH_3_)C(O)O-); ^13^C-NMR (CDCl_3_, δ, ppm): 17.1 (-OCH(**C**H_3_)C(O)O-), 20.9 (-CH(**C**H_3_)C(O)OH), 67.1 (-C**H(**CH_3_)OH, end group), 69.0 (-O**C**H(CH_3_)C(O)O**-**), 169.6 (-**C**(O)O-); FTIR (KBr, cm^-1^): 2,997 (υ_as_CH_3_), 2,947 (υ_s_CH_3_), 2,882 (υCH), 1,760 (υC=O), 1,452 (δ_as_CH_3_), 1,348-1,388 (δ_s_CH_3_), 1,368-1,360 (δ_1_CH+δ_s_CH_3_), 1,315-1,300 (δ_2_CH), 1,270 (δCH + υCOC), 1,215-1,185 (υ_as_COC + r_as_CH_3_), 1,130 (r_as_CH_3_), 1,100-1,090 (υ_s_COC), 1,045 (υC-CH_3_), 960-950 (rCH_3_ + υCC), 875-860 (υC-COO), 760-740 (δC=0), 715-695 (γC=O), 515 (δ_1_C-CH_3_ + δCCO), 415 (δCCO), 350 (δ_2_C-CH_3_ + δCOC), 300-295 (δCOC + δ_2_C-CH_3_), 240 (τCC).

*Norfloxacin*: ^1^H NMR (CDCl_3_, δ , ppm): 1,61; 3,14; 3,31; 4,33; 6,84; 8,07; 8,69; (DMSO-d_6_, δ , ppm): 1,40; 2,88; 3,21; 4,56; 7,13; 7,87; 8,93; ^13^C-NMR (CDCl_3_, δ, ppm): 14,69; 46,07; 49,97; 51,30; 103,87; 108,60; 113,21; 137,34; 147,29; 152,12; 155,46; 167,50; 177,26; FTIR (KBr, cm^-1^): 3,470 (υOH), 1,710 (υCO), 1,624 (υC=C i C=N), 1,452 (δCH_2_ i ωCH_2_), 1,194 (δCH, γCH i υC-O), 1,102 (rings), 801 (υC-N i δCH_2_).

*Norfloxacin - PUR (oligo(ethylene adipate)diol/1,6-hexane diisocyanate)*: ^1^H NMR (DMSO**-**d_6_, δ , ppm): 1.15–1.65 {(-O(O)CNHCH_2_C**H_2_**CH_2_CH_2_C**H_2_**CH_2_NHC(O)-), (-O(O)CNHCH_2_CH_2_C**H_2_**C**H_2_**CH_2_CH_2_NHC(O)-), (-OC(O)CH_2_C**H_2_**C**H_2_**CH_2_C(O)OCH_2_CH_2_-), (-C**H_3_**, NOR)}, 2.31 {-CH_2_C**H_2_**C(O)O-}, 2.85–3.05 {(-NH-C**H_2_**-, NOR), (‑O(O)CNHC**H_2_**CH_2_CH_2_CH_2_CH_2 _C**H_2_**NHC(O)-)}, 3.15 {-C**H_2_**-N, NOR}, 3.62 {-CH_2_C**H_2_**NHC(O)-N(NOR)}, 3.68 {-CH_2_C**H_2_**NHC(O)-C(NOR)}, 4.03 {-OC**H_2_**CH_2_CH_2_CH_2_C(O)-}, 4.11 {-NHC(O)OC**H_2_**CH_2_-}, 4.31 {N-C**H_2_**CH_3_, NOR}, 5.72 {{-CH_2_CH_2_N**H**C(O)-N(NOR)}, 6.59 {-CH_2_CH_2_N**H**C(O)-C(NOR)}, 7.22, 7.92, 9.02 {-C**H**(Ar), NOR}; ^13^C-NMR (DMSO-d_6_, δ , ppm): 22.3 {-O(O)CNHCH_2_CH_2_**C**H_2_**C**H_2_CH_2_CH_2_NHC(O)-}, 25.2 {‑**C**H_2_CH_2_C(O)O-}, 28.8 {-O(O)CNHCH_2_**C**H_2_CH_2_CH_2_**C**H_2_CH_2_NHC(O)-}, 32.9 {-CH_2_**C**H_2_C(O)-}, 40.7 {-O(O)CNH**C**H_2_CH_2_CH_2_CH_2_CH_2_**C**H_2_NHC(O)-}, 64.3 {-C(O)O**C**H_2_CH_2_-}, 172.9 {-CH_2 _**C**(O)O‑}, 173.4 {-O(O)**C**NHCH_2_CH_2_CH_2_CH_2_CH_2_CH_2_NH**C**(O)-} and 14.3, 45.6, 49.0, 50.8, 105.8, 106.8, 110.7, 137.8, 146.5, 148.9, 151.0, 154.5, 167.0, 176.8 {NOR fragments}; FTIR (KBr, cm^-1^):**1**,175 (*υ_s_*COC), 1,192 (*υ*OC-O), 1,243 (*υ*_as_COC), 1,455 (δCH_2 _and ωCH_2_, NOR), 1,627 (νC=C and νC=N, NOR), 1,729 (*υ*C=O), 2,862 (*υ*_s_CH_2_), 2,854 (ν_as_CH_3_), 2,931 (ν_as_CH_2_), 3,334 (νNH, urethane group).

*Norfloxacin - PUR (oligo(ε-caprolactone)diol/1,6-hexane diisocyanate)*: ^1^H NMR (DMSO-d_6_, δ , ppm): 1.15–1.45 {(-O(O)CNHCH_2_C**H_2_**CH_2_CH_2_C**H_2_**CH_2_NHC(O)-), (-O(O)CNHCH_2_CH_2_C**H_2_**C**H_2_**CH_2_CH_2_NHC(O)-), (-OCH_2_CH_2_C**H_2_**CH_2_CH_2_C(O)-)}, 1.50–1.65 {(-C**H_3_**, NOR), (-OCH_2_C**H_2_**CH_2_C**H_2_**CH_2_C(O)-)}, 2.31 {-OCH_2_CH_2_CH_2_CH_2_C**H_2_**C(O)-}, 2.85–3.05 {(-NH-C**H_2_**-, NOR), (-O(O)CNHC**H_2_**CH_2_CH_2_CH_2_CH_2_C**H_2_**NHC(O)-)}, 3.15 {-C**H_2_**-N, NOR}, 3.62 {-CH_2_C**H_2_**NHC(O)-N(NOR)}, 3.68 {-CH_2_C**H_2_**NHC(O)-C(NOR)}, 4.03 {-OC**H_2_**CH_2_CH_2_CH_2_CH_2_C(O)-}, 4.11 {-NHC(O)OC**H_2_**CH_2_-}, 4.31 {N-C**H_2_**CH_3_, NOR}, 5.72 {{-CH_2_CH_2_N**H**C(O)-N(NOR)}, 6.59 {-CH_2_CH_2_N**H**C(O)-C(NOR)}, 7.22, 7.92, 9.02 {-C**H**(Ar), NOR}; ^13^C-NMR (DMSO-d_6_, δ , ppm): 22.3 {-O(O)CNHCH_2_CH_2_**C**H_2_**C**H_2_CH_2_CH_2_NHC(O)-}, 24.6 {-OCH_2_CH_2_**C**H_2_CH_2_CH_2_C(O)-}, 25.6 {‑OCH_2 _**C**H_2_CH_2_**C**H_2_CH_2_C(O)-}, 28.8 {-O(O)CNHCH_2_**C**H_2_CH_2_CH_2_**C**H_2_CH_2_NHC(O)-}, 34.08 {‑OCH_2_CH_2 _CH_2_CH_2_**C**H_2_C(O)-}, 40.7 {-O(O)CNH**C**H_2_CH_2_CH_2_CH_2_CH_2_**C**H_2_NHC(O)-}, 64.3 {‑O**C**H_2_CH_2_CH_2 _CH_2_CH_2_C(O)-}, 172.9 {-OCH_2_CH_2_CH_2_CH_2_CH_2_**C**(O)O-}, 173.4 {‑O(O)**C**NHCH_2_CH_2_CH_2_CH_2_CH_2 _CH_2_NH**C**(O)-} and 14.3, 45.6, 49.0, 50.8, 105.8, 106.8, 110.7, 137.8, 146.5, 148.9, 151.0, 154.5, 167.0, 176.8 {NOR fragments}; FTIR (KBr, cm^-1^):1,170 (*υ_s_*COC), 1,190 (*υ*OC-O), 1,240 (*υ*_as_COC), 1,452 (δCH_2 _and ωCH_2_, NOR), 1,624 (νC=C and νC=N, NOR), 1,727 (*υ*C=O), 2,865 (*υ*_s_CH_2_), 2,856 (ν_as_CH_3_), 2,933 (ν_as_CH_2_), 3,322 (νNH, urethane group).

*Norfloxacin - PUR (oligolactide diol/1,6-hexane diisocyanate)*: ^1^H NMR (DMSO**-**d_6_, δ, ppm): 1.15–1.45 {(-O(O)CNHCH_2_C**H_2_**CH_2_CH_2_C**H_2_**CH_2_NHC(O)-), (‑O(O)CNHCH_2_CH_2_C**H_2_**C**H_2_**CH_2_CH_2_NH C(O)-}, 1.50–1.65 {(-C**H_3_**, NOR), (-CH(C**H**_3_)}, 2.85–3.05 {(-NH-C**H_2_**-, NOR), (‑O(O)CNHC**H_2 _**CH_2_CH_2_CH_2_CH_2_C**H_2_**NHC(O)-)}, 3.15 {-C**H_2_**-N, NOR}, 3.32 {-(CH_3_)C**H**NHC(O)-N(NOR)}, 3.41 {‑(CH_3_)C**H**NHC(O)-C(NOR)}, 3.86 {-NHC(O)O(CH_3_)C**H**-}, 4.31 {N-C**H_2_**CH_3_, NOR}, 5.15–5.35 {(-C**H**(CH3)-), (-(CH_3_)C**H**N**H**C(O)-N(NOR)}, 6.59 {-CH_2_CH_2_N**H**C(O)-C(NOR)}, 7.22, 7.92, 9.02 {‑C**H**(Ar), NOR}; ^13^C-NMR (DMSO-d_6_, δ, ppm): 16.8 {-CH(**C**H3)}, 22.5 {‑O(O)CNHCH_2_CH_2_**C**H_2_**C**H_2_CH_2_CH_2_NHC(O)-}, 28.9 {-O(O)CNHCH_2_**C**H_2_CH_2_CH_2_**C**H_2_CH_2_NHC (O)-}, 40.9 {-O(O)CNH**C**H_2_CH_2_CH_2_CH_2_CH_2_**C**H_2_NHC(O)-}, 69.2 {-**C**H(CH3)-}, 169.80 {‑**C**(O) O‑}, 173.5 {-O(O)**C**NHCH_2_CH_2_CH_2_CH_2_CH_2_CH_2_NH**C**(O)-} and 14.2, 45.4, 49.0, 50.7, 105.7, 106.9, 110.5, 137.5, 146.4, 148.7, 151.0, 154.4, 167.0, 176.6 {NOR fragments}; FTIR (KBr, cm^-1^):240 (τCC), 300-295 (δCOC + δ_2_C-CH_3_), 350 (δ_2_C-CH_3_ + δCOC), 415 (δCCO), 515 (δ_1_C-CH_3_ + δCCO), 715-695 (γC=O), 760-740 (δC=O), 875-860 (υC-COO), 960-950 (rCH_3_ + υCC), 1,045 (υC-CH_3_), 1,100-1,090 (υ_s_COC), 1,130 (r_as_CH_3_), 1,215-1,185 (υ_as_COC + r_as_CH_3_), 1,270 (δCH + υCOC), 1,315-1,300 (δ_2_CH), 1,360-1,370 (δ_1_CH+δ_s_CH_3_), 1,350-1,390 (δ_s_CH_3_), 1,450 (δ_as_CH_3_ and δCH_2 _and ωCH_2_, NOR), 1,625 (νC=C and νC=N, NOR), 1,760 (υC=O), 2,880 (υCH), 2,995 (υ_as_CH_3_), 2,950 (υ_s_CH_3_).

## 4. Conclusions

It has been proved that the ring-opening polymerization of L-LA, D,L-LA and ECL in the presence of CE is a very efficient method of the synthesis of low-molecular weight polyesters, terminated by hydroxyl groups. Polymerization at 160 °C in bulk produced polymers with a high yield (even ca. 90% in some cases). It has been then shown that oligoesters prepared in this way may be applied for the synthesis of polyurethane conjugates of NOR. The synthesis of those conjugates was done in three steps. First, the ring-opening homo- or copolymerization of L-LA, D,L-LA and ECL in the presence of CE and EG was carried out. In the second step, the prepolymer was obtained. In the final step, the reaction of the prepolymer with NOR was performed. The possibility of using the obtained conjugates as prodrugs of norfloxacin is currently in progress. The latter application requires yet careful, subsequent *in vitro * and *in vivo* examinations. We believe that the obtained polyurethane conjugates of norfloxacin are good potential candidates for carriers in drug delivery systems.

## References

[1] Jagur-Grodzinski J. (1999). Biomedical application of functional polymers. React. Funct. Polym..

[2] Uhrich K.E., Cannizzaro S.M., Langer R.S., Shakesheff K.M. (1999). Polymeric systems for controlled drug release. Chem. Rev..

[3] Veronese F.M., Morpurgo M. (1999). Bioconjugation in pharmaceutical chemistry. Farmaco.

[4] Hoste K., De Winne K., Schacht. (2004). Polymeric prodrugs. Int. J. Pharm..

[5] Ouchi T., Ohya Y. (1995). Macromolecular prodrugs. Prog. Polym. Sci..

[6] Garnett M.C. (2001). Targeted drug conjugates: Principles and progress. Adv. Drug Del. Rev..

[7] Merkli A., Tabatabay C., Gurny R., Heller J. (1998). Biodegradable polymers for the controlled release of ocular drugs. Prog. Polym. Sci..

[8] Järvinen T., Järvinen K. (1996). Prodrugs for improved ocular delivery. Adv. Drug Del. Rev..

[9] Khandare J., Minko T. (2006). Polymer-drug conjugates: Progress in polymeric prodrugs. Prog. Polym. Sci..

[10] Sobczak M., Olędzka E., Kolodziejski W., Kuźmicz R. (2007). Pharmaceutical application of polymers. Polimery.

[11] Olędzka E., Sobczak M., Kołodziejski W.L. (2007). Polymers in medicine -review of recent studies. Polimery.

[12] Król P. (2007). Synthesis methods, chemical structures and phase structures of linear polyurethanes. Properties and applications of linear polyurethanes in polyurethane elastomers, copolymers and ionomers. Prog. Polym. Sci..

[13] Oertel G. (1994). Polyurethane Handbook.

[14] Wirpsza Z. (1993). Polyurethanes: Chemistry, Technology and Application.

[15] Kuran W., Sobczak M., Listoś T., Dębek C., Florjańczyk Z. (2000). New route to oligocarbonate diols suitable for the synthesis of polyurethane elastomers. Polymer.

[16] Rokicki G., Piotrowska A. (2002). A new route to polyurethanes from ethylene carbonate, diamines and diols. Polymer.

[17] Neffgen S., Keul H., Höcker H. (1997). Cationic ring-opening polymerization of trimethylene urethane: A mechanistic study. Macromolecules.

[18] Kusan J., Keul H., Höcker H. (2001). Cationic ring-opening polymerization of tetramethylene urethane. Macromolecules.

[19] Albertsson A.C., Varma I.V. (2003). Recent developments in ring opening polymerization of lactones for biomedical applications. Biomacromolecules.

[20] Florjańczyk Z., Plichta A., Sobczak M. (2006). Ring opening polymerization initiated by methylaluminoxane/AlMe3 complexes. Polymer.

[21] Marcilla R., de Geus M., Mecerreyes D., Duxbury C.J., Koning C.E., Heise A. (2006). Enzymatic polyester synthesis in ionic liquids. Eur. Polym. J..

[22] He F., Li S., Garreau H., Vert M., Zhuo R. (2005). Enzyme-catalyzed polymerization and degradation of copolyesters of ε-caprolactone and γ-butyrolactone. Polymer.

[23] Duda A., Biela T., Kowalski A., Lubiszowski J. (2005). Amines as (co)initiators of cyclic esters' polymerization. Polimery.

[24] Martin E., Dubois P., Jerome R. (2003). “In Situ” Formation of Yttrium Alkoxides: A Versatile and Efficient Catalyst for the ROP of ε-Caprolactone. Macromolecules.

[25] Storey R.F., Sherman J.W. (2002). Kinetics and mechanism of the stannous octoate-catalyzed bulk polymerization of ε-caprolactone. Macromolecules.

[26] Kobayashi S., Uyama H., Kimura S. (2001). Enzymatic polymerization. Chem. Rev..

[27] Kowalski A., Duda A., Penczek S. (2000). Kinetics and mechanism of cyclic esters polymerization initiated with tin(II) octoate. 3. Polymerization of L,L-dilactide. Macromolecules.

[28] Divakar S. (2004). Porcine pancreas lipase catalyzed ring-opening polymerization of ε-Caprolactone. J. Macromol. Sci. Pure Appl. Chem..

[29] Namekawa S., Suda S., Uyama H., Kobayashi S. (1999). Lipase-catalyzed ring-opening polymerization of lactones to polyesters and its mechanistic aspects. Int. J. Biol. Macromol..

[30] Sobczak M., Kolodziejski W. (2009). Polymerization of cyclic esters initiated by carnitine and tin (II) octoate. Polymerization of cyclic esters initiated by carnitine and tin (II) octoate. Molecules.

[31] Sobczak M., Olędzka E., Kołodziejski W.L. (2008). Polymerization of cyclic esters using aminoacid initiators. J. Macromol. Sci. A.

[32] Wang C., Li H., Zhao X. (2004). Ring opening polymerization of l-lactide initiated by creatinine. Biomaterials.

[33] Hu W., Zhou W., Xia C., Wen X. Synthesis and anticancer activity of thiosemicarbazones. Bioorg. Med. Chem. Lett..

[34] Jeon H.-J., Jeong Y.-I., Jang M.-K., Park Y.-H., Nah J.-W. (2000). Effect of solvent on the preparation of surfactant-free poly(DL-lactide-co-glycolide) nanoparticles and norfloxacin release characteristics. Int. J. Pharm..

[35] Sobczak M., Witkowska E., Olędzka E., Kolodziejski W. (2008). Synthesis and Structural analysis of polyester prodrugs of norfloxacin. Molecules.

[36] Górna K., Gogolewski S. (2003). Molecular stability, mechanical properties, surface characteristics and sterility of biodegradable polyurethanes treated with low-temperature plasma. Polym. Degrad. Stabil..

